# Paediatric spinal cord infarction—a review of the literature and two case reports

**DOI:** 10.1007/s00381-016-3295-8

**Published:** 2016-11-26

**Authors:** Asim Sheikh, Daniel Warren, Anne-Marie Childs, John Russell, Mark Liddington, Velupandian Guruswamy, Paul Chumas

**Affiliations:** 1 Department of Neurosurgery, Leeds General Infirmary, Great George Street, Leeds, UK; 2 Department of Radiology, Leeds, UK; 3 Department of Paediatrics, Leeds, UK; 4 Department of Maxillofacial surgery, Leeds, UK; 5 Department of plastic surgery, Leeds, UK; 6 Department of Anaesthesia, Leeds, UK

**Keywords:** Spinal cord, Infarction, Ischaemic

## Abstract

Ischemic spinal cord infarction is rare in the paediatric population, and when it does occur, it is usually associated with traumatic injury. Other potential causes include congenital cardiovascular malformations, cerebellar herniation, thromboembolic disease and infection. Magnetic resonance imaging (MRI) findings can be subtle in the early evaluation of such patients. The outcome is variable and depends on the level and extent of the spinal cord infarct and subsequent rehabilitation. Here, we present two cases of ischemic spinal cord infarction in children.

## Introduction

Although spinal cord infarction in children is rare, it can lead to long lasting deficits and the need for ongoing care. It is fortunately a rare occurrence. It can be difficult to diagnose clinically in the very young and can be mistaken for myelopathy; MRI findings can be difficult to interpret due to their subtle nature in the hyperacute phase. We present two cases of ischemic spinal cord infarction in children.

## Case 1

**Fig. 1:**
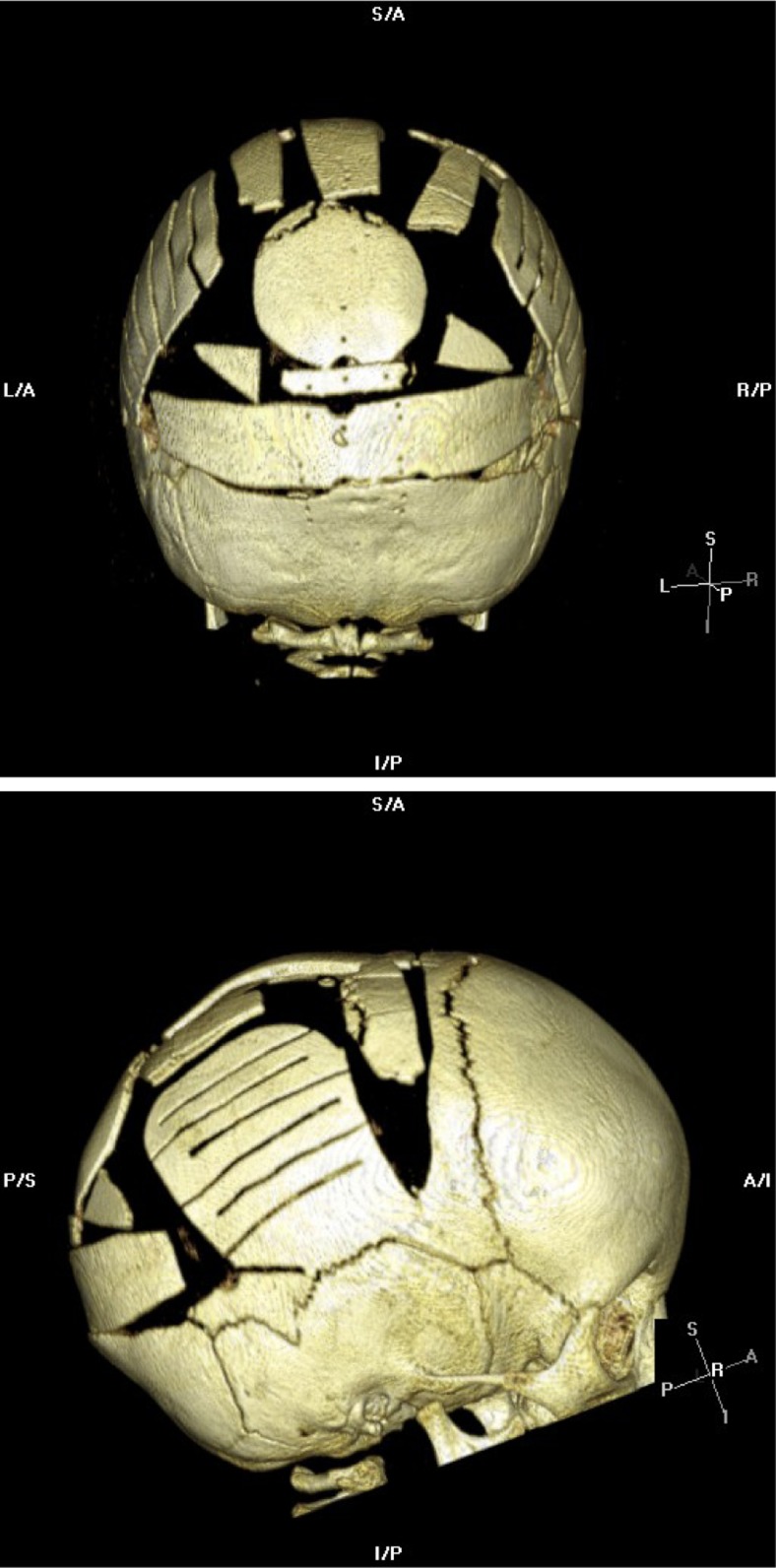
CT Multiplanar reconstruction (MPR) demonstrating the postoperative remodelled calvarium following craniosynostosis surgery

A 9-month-old fit and healthy boy underwent posterior 2/3 calvarial remodelling surgery for sagittal synostosis (Fig. 1). This was carried out in the supine position while the head was supported on a horse-shoe head rest with the head well flexed. A gel pad for was used for chin rest. This is the usual patient position for such procedures in our institution. The mean arterial pressure was monitored throughout the procedure continuously via arterial cannulation, and there was no period of hypotension or excessive blood loss noted during surgery—with the lowest recorded intraoperative haemoglobin (Hb) being 82 g/L. No ventilation problems were encountered during the procedure. Immediately postoperatively, the Hb was noted to be 70 g/L and he was transfused 1 unit of red blood cells.

**Fig. 2:**
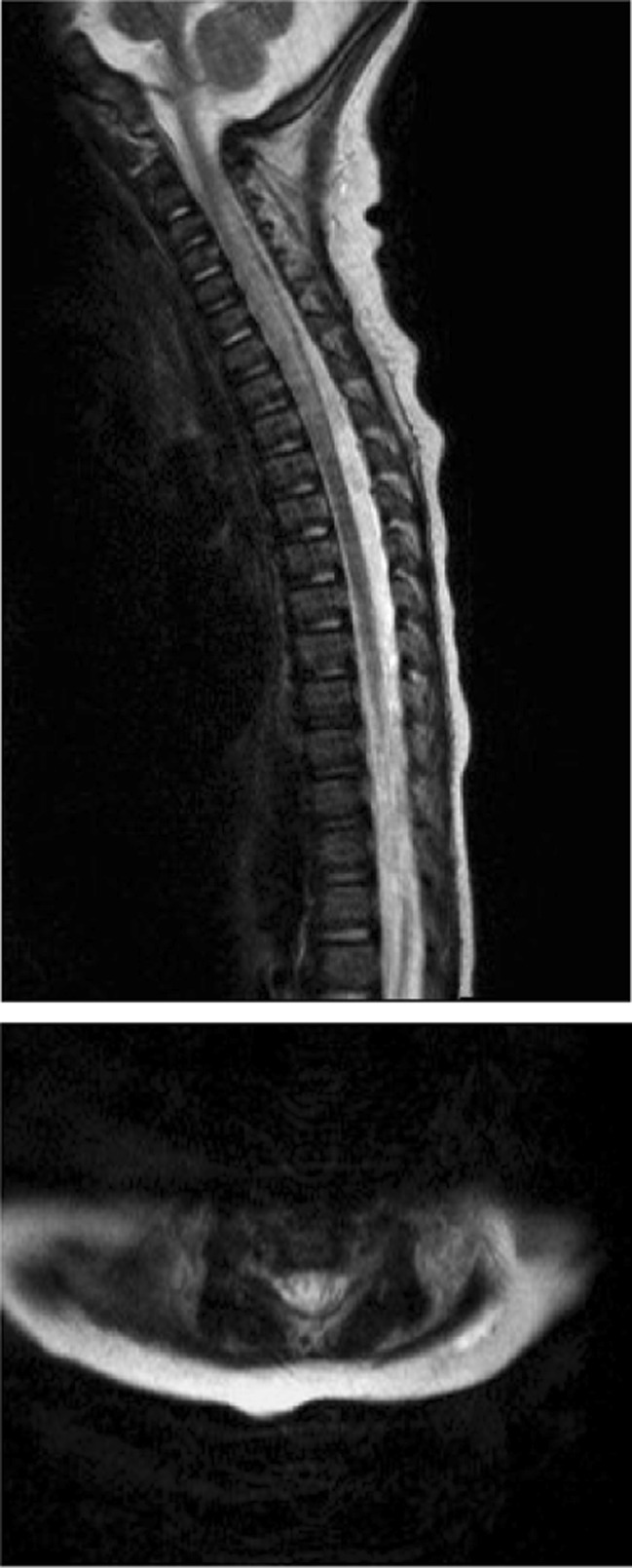
Initial MRI imaging obtained 2 days following surgical intervention. Imaging assessment was significantly degraded by motion artefact; however, the T2 imaging clearly shows abnormal cord expansion and T2 signal change extending between C2/3 and T2

**Fig. 3:**
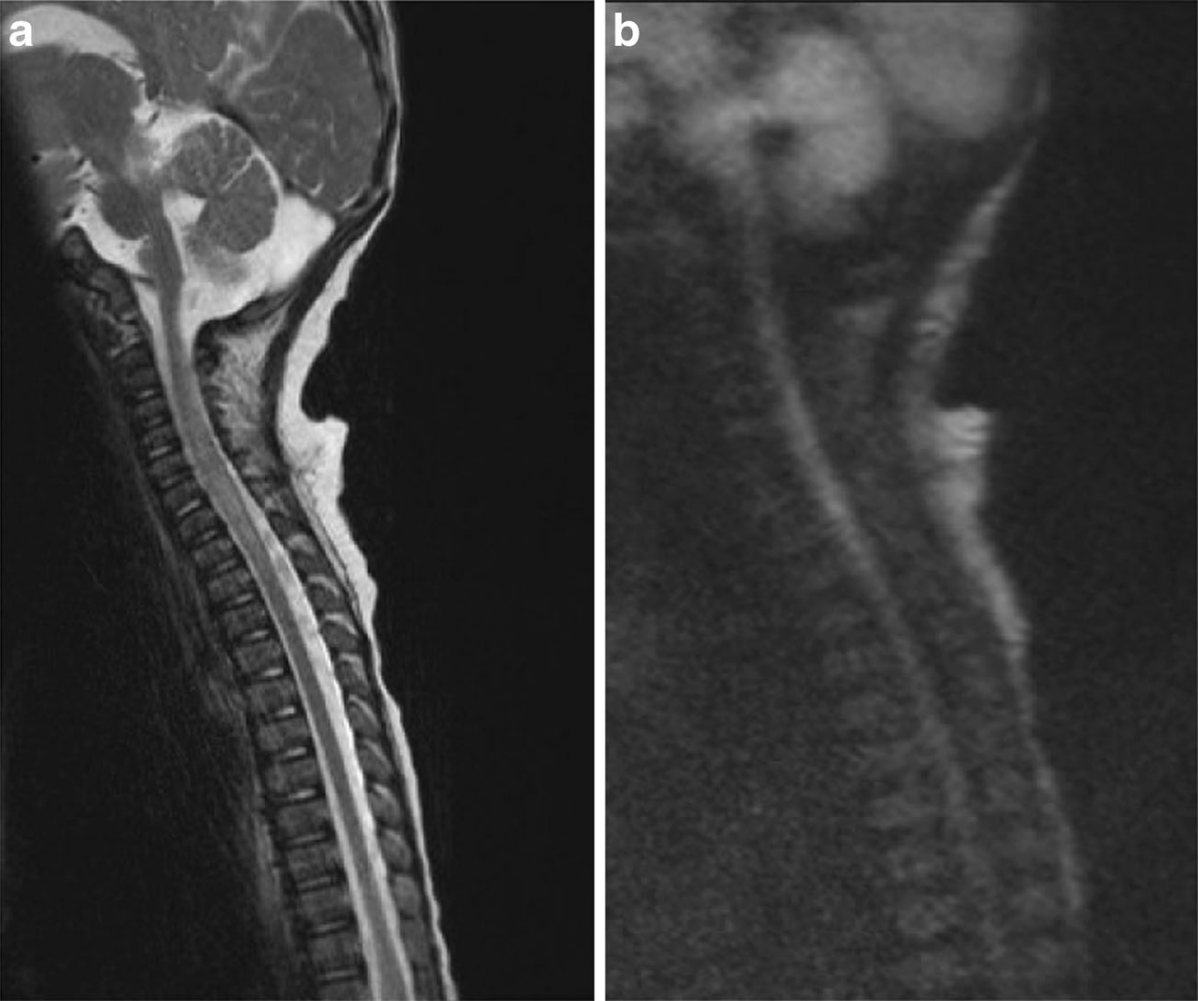
Repeat MRI assessment 2 days later confirmed the cord signal abnormality on standard T2 imaging (**a**) and showed concomitant apparent restriction on HASTE diffusion weighted imaging (DWI) (**b**) supporting a suspected acute ischaemic insult

On second postoperative day, it was apparent that he was not moving his legs and could only flex his arms to the mid chest. A CT head scan did not reveal any acute intracranial pathology to explain his clinical findings. Subsequent MRI of his brain and spinal cord revealed high T2 intramedullary signal between C2 and T2, with concomitant expansion of the cervical spinal cord (Figs. 2 and 3). MRI brain imaging was normal. No traumatic injury was encountered peri-operatively, and this was evident from the lack of signal changes on the MRI in the vertebrae and cervical spinal ligaments. There was no suggestion of any viral illness or evidence of myelitis. A profound hypotensive episode was neither encountered clinically nor suggested on the radiological assessment. There was no evidence of cerebral hypoperfusion insult.

Cardiac imaging did not show any abnormality and coagulation profile was found to be normal for his age. This included standard coagulation profile with prothrombin time, APTT ratio, derived fibrinogen and INR. This also included PT 20210 gene screen for FII gene mutation, lupus anticoagulant, thrombophilia screen, antiphospholipid antibodies including cardiolipin antibody and B2-glycoprotein IgG and lastly, factor V Leiden mutation. This flaccid paralysis lasted for up to 12 days after surgery when he was seen to move his legs. The left arm regained more movement first, followed by his right arm in the initial postoperative period. An intense rehabilitative physiotherapy regimen was commenced. By 28th postoperative day, he was noted to have MRC grade 4 power in the right arm, grade 3 in the left arm and grade 2 in both legs. Truncal tone improved by day 33 postop, when he started sitting upright with support. A repeat MRI at 6 weeks post surgery showed improvement in cord swelling but with persistent signal change secondary to myelomalacia. The images were degraded due to motion artefact and are not included here.

He continued to engage in a physiotherapy programme, and after initially being supported with a rigid spinal brace, he was subsequently provided with a lycra corset. Follow-up review 21 months after this event revealed significantly improved right arm function. He was able to sit and stand unsupported but required significant support while walking.

## Case 2

**Fig. 4:**
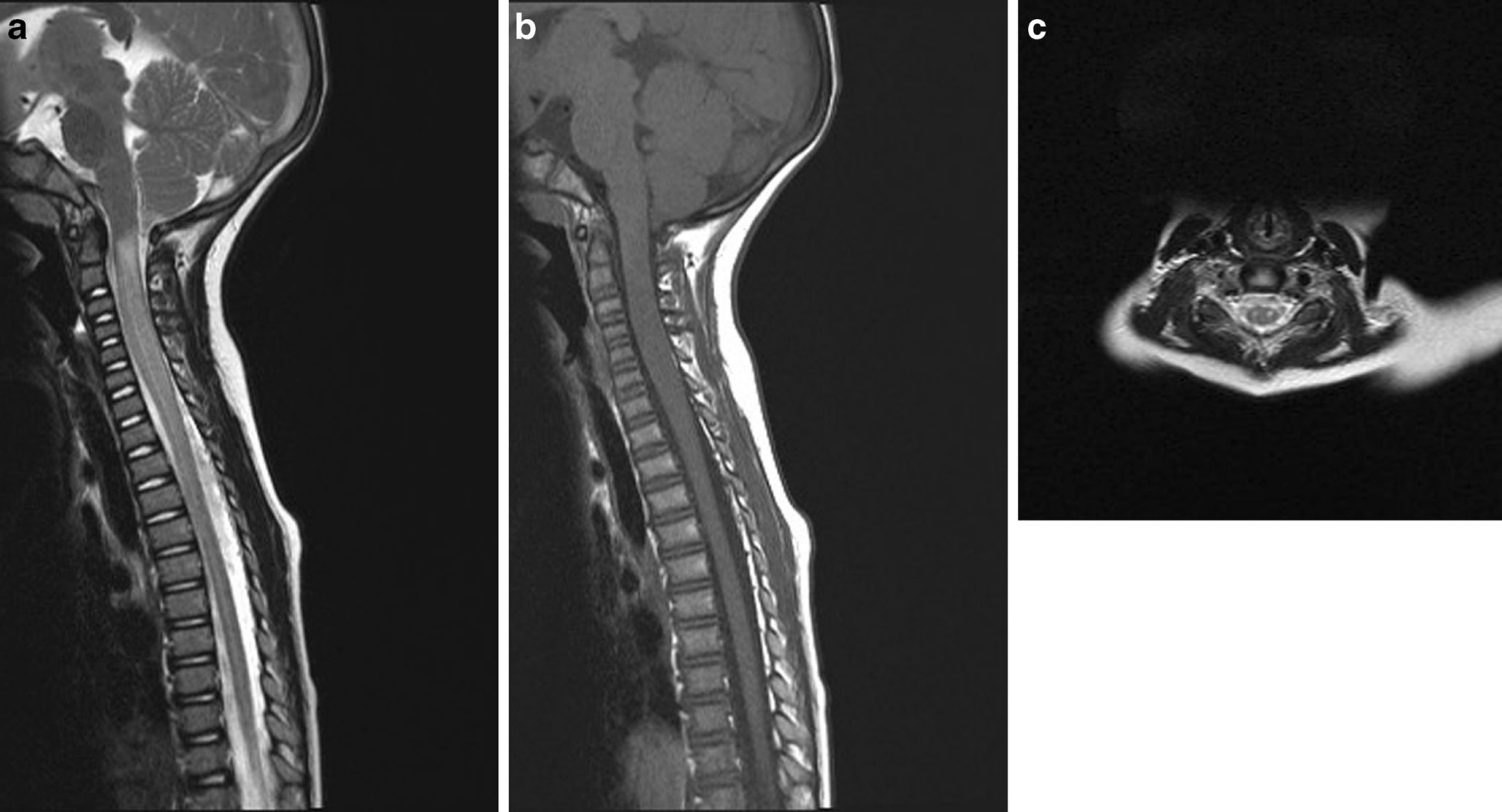
**a** T2 and **b** T1 Sagittal MRI of the cervical spine demonstrating diffuse swelling (**a**, **b**) and increased T2 signal (**a**) of the cervical cord substance. Confirmed on axial T2 assessment at the C5 level (**c**)

**Fig. 5:**
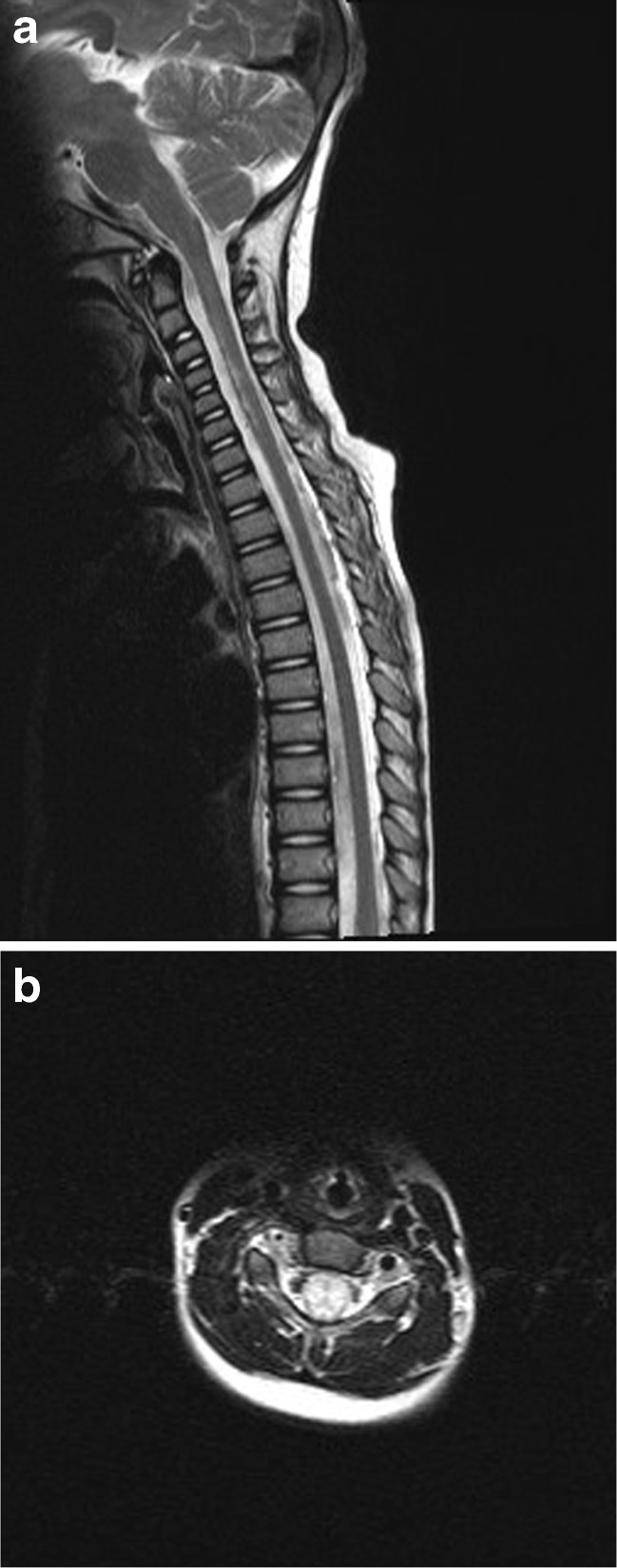
Follow-up MRI at 6 weeks confirms interval volume loss and established myelomalacia on sagittal (**a**) and axial (**b**) T2 imaging

The second patient was a 2-year-old girl, who while playing with her father and siblings, fell from kneeling position onto a soft mattress, head first. Within minutes of this fall, she developed left sided weakness followed by flaccid paresis of all four limbs which continued to worsen. She was brought to our institution where MRI brain and spine imaging was performed. The MRI brain was normal but her cervical spine assessment demonstrated significant cord expansion with increased intramedullary T2 signal abnormality between C1 and C6 (Fig. 4). With a trivial preceding trauma, the initial suspicion was that of transverse myelitis. However, a rapid onset and steady evolution to the point of requiring intermittent positive pressure ventilation was not consistent with natural course of transverse myelitis. She was treated with antimicrobials and steroids in the beginning, until it became apparent subsequently that the cause was vascular insult. A subsequent interval scan 6 weeks after injury showed resolution of the cord swelling but with anterior myelomalacia consistent with the presumed vascular insult (Fig. 5). She continued to make minimal improvement over the course of 6 months after her presentation.

Three years after this event, she remains dependent on ventilation through tracheostomy, only managing few hours a day without ventilatory support and has repeated chest infections. She is mobile in a powered chair, managing the controls with no difficulty. She has good head control, limited independent sitting balance and uses a spinal brace for truncal support. She has functional movement in her right arm with reduced power and some limited distal movements in the left arm. She has some active movement in her left foot. She tolerates standing with full length callipers and support for up to 2 h. She has a neuropathic bladder and bowel.

## Discussion

The cervical spinal cord is known to have an extensive collateral blood supply in order to protect it from ischaemic damage. The spinal cord is supplied by three longitudinal arteries, the anterior spinal artery with a posterior spinal artery anastomosed by the arterial vasocorona [1]. The origin of these vessels is from both vertebral arteries and medullary segmental arteries from the aorta, the largest of which is named as artery of Adamkiewicz [2]. Because of its tenuous supply, the spinal cord is most vulnerable in the mid-thoracic portion. A dramatic drop in blood pressure such as seen in cardiac arrest or excessive blood loss may lead to an infarction at this level [3]. The result can be just as severe as if the spinal cord was severed by a knife [4]. Spinal cord infarction is commoner in adults than children. This has been linked to vascular compromise from atherosclerosis, abdominal aortic surgery and hypotension [5, 6]. Cervical spondylosis is also implicated in reduced cervical vascular supply and has been described following posterior fossa surgery in the sitting position in at least eight cases with age ranges between 4 and 45 years [7, 8].

Being a rare condition, spinal cord infarction in children remains poorly described in the literature. Postmortem examination for infantile deaths within 4 weeks showed ischemic spinal cord damage in 21 out of 900 children examined in the paediatric age group; poor autoregulation of spinal blood flow in premature children is hypothesised as one of the causes. Sladky and Rourke [2] showed 9 out of 900 premature infants to have evidence of spinal cord ischemia on autopsy. Singer et al. reaffirmed this finding in three premature neonates [9]. Achondroplasia is also associated with a higher incidence of spinal cord infarction with three case reports [10] in the literature. This is from presumed spinal stenosis as well as compression at craniocervical junction found in children with achondroplasia. Systemic hypotension as a result of cardiac arrest or intraoperative blood loss can lead to spinal cord infarction. Local shunting due to arteriovenous malformations can cause decreased perfusion and subsequent infarction in a localised segment [11]. Trauma and vascular compromise due to aortic dissection or congenital cardiovascular abnormalities can also lead to infarction [12]. In children, the spinal cord is thought to be less elastic and the vertebral column more flexible. Hence, hyperflexion injuries can cause reactive vasospasm that leads to ischemic insult, as shown by Pang et al. [13, 14]. Cerebellar herniation following lumbar puncture has also been shown to cause spinal vascular compromise [15]. Although spinal cord injury is a well-known complication of surgery performed in a sitting position, it is still very rare to see in paediatric age group [16].

There have been no case reports to our knowledge of spinal cord infarction following calvarial remodelling surgery. The supine position is utilised by many craniofacial teams for calvarial remodelling [4]—and we have used this position for 20 years without incident. Nonetheless, as no other obvious cause can be found for this complication in this patient, we can only assume that some unidentified idiosyncratic factor(s) made our patient vulnerable to surgery in this position—including possibly a combination of mild traction and flexion on the cervical spine. In an attempt to overcome this, we now use a beanbag placed under the child and placed behind the neck for additional support. However, our second case shows that this type of insult can occur after mild trauma—emphasising our continued lack of understanding of these rare events. A review of literature reveals various case reports of spinal cord infarction, described in paediatric patients. The aetiology ranges widely, but a number of other cases have been associated with minor trauma. A summary of these can be seen in Table 1.

**Table 1:** Literature reports of paediatric cases with spinal cord infarction

Author	Year of publication	No of cases	Paediatric cases
Jessica R Nance et al.	2007	2	Non traumatic
Bandyopadhyay S et al.	1999	1	AVM
Brown MS et al.	1988	2	Umbilical artery catheterisation
Aziz EM	1973	1	Umbilical artery catheterisation
Krishnamoorthy KS	1976	1	Umbilical artery catheterisation
Boglino C et al.	1999	1	Thoracic neuroblastoma removal
Castro-Vilanova et al.	1999	4	Aortic surgery complication
Garland H et al.	1966	1	Aortic dissection
Nitta H et al.	1997	1	Sitting position for pineal
Ahmann PA et al.	1975	2	Minor trauma
Choi JU et al.	1986	8	Minor trauma
Ergun A et al.	2003	1	Minor trauma
Cheshire DJ et al.	1977	3	Minor trauma
Riviello JJ et al.	1990	2	Minor trauma
Lenn NJ et al.	1977	1	Minor trauma
Tal Y et al.	1980	3	Post meningitis
Seidman E et al.	1984	1	Oesophageal varices treatment
Moffett KS et al.	1997	1	Post *E. coli* meningitis
Pelser H et al.	1993	2 paeds	6 adults
Naiman J et al.	1961	1	Nucleus pulposus embolism
Yousef OM et al.	1998	1	Nucleus pulposus embolism

Thromboembolic phenomenon has been found to cause spinal cord infarcts as well. This is seen in children with central nervous system infections and autoimmune disorders causing vasculitis. Fibrocartilagenous embolism due to embolisation of nucleus pulposus material is a well-known entity causing acute ischemic changes [17–19]. MRI findings often include changes in intervertebral discs in addition to the usual features of spinal cord infarction [18, 20, 21]. Even with improved imaging techniques, the underlying cause in many children presenting with acute spinal cord infarction remains unclear. A pre-existing vascular anomaly may confer an increased risk of infraction, but spinal vessels are too small to resolve on standard imaging. Venous infarction may also explain MRI signal change. This was a possible mechanism of injury in our second case described.

With the advent of MRI, early diagnosis is now possible in suspected cases. Diffusion weighted imaging can identify ischemic insult within hours of the event [22]. In children, the examination is often limited, however, due to movement and CSF flow artefacts and the relatively smaller cross-sectional area available for examination [22, 23]. It can also be difficult to differentiate spinal cord infarction from myelitis in the early phase [24]. However, as demonstrated by our cases, a high level of suspicion is needed even in trivial trauma, to diagnose vascular insults in children, so they can access timely intervention and effective management strategies.

## References

[1] Walter Hendelman, 2005 Atlas of functional neuroanatomy. CRC Press

[2] Sladky JT, Rorke LB (1986). Perinatal hypoxic/ischemic spinal cord injury. Pediatr Pathol.

[3] Gilles FH, Nag D (1971). Vulnerability of the human spinal cord in transient cardiac arrest. Neurology.

[4] Greensmith AL, Holmes AD, Lo P, Maxiner W, Heggie AA, Meara JG (2008). Complete correction of severe scaphocephaly: the Melbourne method of total vault remodeling. Plast Reconstr Surg.

[5] Brewer LA, Fosburg RG, Mulder GA, Verska JJ (1972). Spinal cord complications following surgery for coarctation of the aorta. A study of 66 cases. J Thorac Cardiovasc Surg.

[6] Cheshire WP, Santos CC, Massey EW, Howard JF (1996). Jr spinal cord infarction: etiology and outcome. Neurology.

[7] Martinez JF, Almagro MJ, Izura V, Serrano C, Ruiz-Espejo AM, Sanchez-Del-Rincon I (2009). Cervical spinal cord infarction after posterior fossa surgery: a case base update. Childs Nerv Syst.

[8] Sandson TA, Friedman JH Spinal cord infarction: report of 8 cases and review of the literature 2677596

[9] Singer R, Joseph K, Gilai AN, Meyer S (1991). Nontraumatic, acute neonatal paraplegia. J Pediatr Orthop.

[10] Wieting JM, Krach LE (1994). Spinal cord injury rehabilitation in a pediatric achondroplastic patient: case report. Arch Phys Med Rehabil.

[11] Riche MC, Modenesi-Freitas J, Djindjian M, Merland JJ (1982). Arteriovenous malformations (avm) of the spinal cord in children. A review of 38 cases. Neuroradiology.

[12] Hasegawa M, Yamashita J, Yamashima T, Ikeda K, Fujishima Y, Yamazaki M (1993) Spinal cord infarction associated with primary antiphospholipid syndrome in a young child. Case report. J Neurosurg 79:446–450 10.3171/jns.1993.79.3.0446 8360745

[13] Pang D (2004). Spinal cord injury without radiographic abnormality in children, 2 decades later. Neurosurgery.

[14] Pang D, Wilberger JE (1982). Jr spinal cord injury without radiographic abnormalities in children. J Neurosurg.

[15] Norman MG (1982). Respiratory arrest and cervical spinal cord infarction following lumbar puncture in meningitis. Can J Neurol Sci.

[16] Nitta H, Yamashita J, Nomura M, Igarashi N (1997). Cervical spinal cord infarction after surgery for a pineal region choriocarcinoma in the sitting position: case report. Neurosurgery.

[17] Davis GA, Klug GL (2000). Acute-onset nontraumatic paraplegia in childhood: fibrocartilaginous embolism or acute myelitis?. Childs Nerv Syst.

[18] Han JJ, Massagli TL, Jaffe KM (1989). Fibrocartilaginous embolism—an uncommon cause of spinal cord infarction: case report and review of the literature. Arch Phys Med Medicine (Baltimore).

[19] Tosi L, Rigoli G, Beltramello A (1996). Fibrocartilaginous embolism of the spinal cord: a clinical and pathogenetic reconsideration. J Neurol Neurosurg Psychiatry.

[20] Raghavan A, Onikul E, Ryan MM, Prelog K, Taranath A, Chennapragada M (2004). Anterior spinal cord infarction oweing to possible fibrocartilaginous embolism. Pediatr Radiol.

[21] Yousef OM, Appenzeller P, Kornfeld M (1998). Fibrocartilagenous embolism: an unusual cause of spinal cord infarction. Am J Forensic Med Pathol.

[22] Kuker W, Weller M, Klose U, Krapf H, Dichgans J, Nagele T (2004) Diffusion-weighted MRI of spinal cord infarction—high resolution imaging and time course of diffusion abnormality. J Neurol 251:818–824 10.1007/s00415-004-0434-z 15258783

[23] Thron AK, 1988 Vascular anatomy of the spinal cord: neuroradiological investigations and clinical syndromes. Chapter V. New York: Springer-Verlag Wien. Postmortem angiography and microangiography of spinal cord vessels; pp. 13–64

[24] Masson C, Pruvo JP, Meder JF, Cordonnier C, Touze E, De La Sayette V, Giroud M, Mas JL, Leys D (2004). Spinal cord infarction: clinical and magnetic resonance imaging findings and short term outcome. J Neurol Neurosurg Psychiatry.

